# Lamivudine Inhibits the Replication of ALV-J Associated Acutely Transforming Virus and its Helper Virus and Tumor Growth *In vitro* and *In vivo*

**DOI:** 10.3389/fmicb.2015.01306

**Published:** 2015-12-01

**Authors:** Yixin Wang, Shuzhen Xu, Sifei Li, Hongqin Su, Shuang Chang, Yang Li, Xiaolong Sun, Peng Zhao, Zhizhong Cui

**Affiliations:** College of Animal Science and Veterinary Medicine, Shandong Agricultural UniversityTai’an, China

**Keywords:** avian leukosis virus, acutely transforming, acute fibrosarcoma, lamivudine, antiviral

## Abstract

To study the antiviral effects of lamivudine on avian leukosis virus subgroup J (ALV-J) and its inhibitory effect on the growth of fibrosarcomas caused by acute transforming avian leukosis virus, a series of experiments were performed in chicken embryo fibroblast cultures and 1-day-old chickens inoculated with an acutely transforming viral stock Fu-J (SDAU1005). This stock was prepared from an acutely fibrosarcoma of field cases in chicken farms and contained both the replication-defective virus Fu-J carrying *v-fps* oncogene and its helper virus ALV-J strain SDAU1005. The results from three different assays in cell cultures demonstrated the significant inhibitory effect of lamivudine on the replication of both SDAU1005 and Fu-J viruses. Furthermore, the effect was dose dependent in the concentration range of 1–4 μg/ml. In chicken experiments, lamivudine could decrease the viral loads of SDAU1005 and Fu-J in the plasma of inoculated chickens, delay the appearance of acute sarcomas, and decrease chicken mortality in the early stage. This model may be used to directly evaluate the inhibitory effects of lamivudine on such tumors and to understand the relationship between the replication-defective virus and its helper virus while also assessing tumor processes.

## Introduction

Avian leukosis virus (ALV) is a member of the Retroviridae family and can cause neoplastic disease in chickens by both vertical and horizontal transmission. It can be divided into 10 subgroups from A to J according to the viral envelope glycoprotein and neutralization test (Chesters et al., 2002). ALV-J was first isolated from the UK and mainly caused myeloid leukosis and hemangioma in broilers and layers in 1991 (Payne et al., 1991, 1992; Bai et al., 1995). The epidemic of ALV-J worldwide in the following years caused enormous economic losses for the poultry industry and attracted significant government attention in many countries around the world. The infection and spread of ALV-J has been controlled effectively in western countries through advanced technology and management experience (Payne and Nair, 2012). However, it remains a significant challenge to monitor and control ALV-J due to the late start and huge scale of chicken flocks in China, although remarkable progress has been achieved during the last decade (Gao et al., 2010). Infection of ALV-J is ubiquitous in almost all types of chicken especially in Chinese local breeds (Sun et al., 2010; Lai et al., 2011; Li et al., 2013; Wang et al., 2013a; Dong et al., 2015).

At present, effective therapeutics and preventive vaccines are unavailable because ALV-J has a high mutation rate, and the host immune responses cannot resist viral infections effectively (Hwang and Wang, 2002; Williams et al., 2004; Wang and Cui, 2006). Effective antiviral agents can prevent the virus from replicating, and the rational use of drugs can speed up the process of ALV-J surveillance and control. However, the study of anti-ALV drugs has just commenced, and it is both time consuming and costly to select effective drugs using ALV-J infection models. This is due to the long period of at least 20 weeks to induce tumors in chickens by classical ALV-J. Lamivudine is a type of nucleoside derivative antiviral drug with a competitive inhibitory effect on viral DNA synthesis and extension (Kewn et al., 1997). It has been shown to function as a nucleoside reverse transcriptase inhibitor and can restrain the replication of human immunodeficiency virus (HIV) by competition binding with cellular nucleotide both *in vitro* and *in vivo* (Soudeyns et al., 1991; Coates et al., 1992; Geleziunas et al., 1993; Tisdale et al., 1993; Gray et al., 1995). As a type of retrovirus, reverse transcriptase also plays a key role in the life cycle of ALV-J, but until now, no research has been conducted to estimate the antiviral effect of lamivudine on ALV.

To explore this area, viral stocks of acutely transforming virus Fu-J (SDAU1005), prepared from fibrosarcoma cell-free filtrate consisting of both the replication-defective virus Fu-J carrying *v-fps* oncogene and its helper virus ALV-J strain SDAU1005, were used to estimate the antiviral effect of lamivudine on ALV-J. Both subcutaneous and intraperitoneal inoculation with viral stocks of acutely transforming virus Fu-J (SDAU1005) could induce acute fibrosarcomas in chickens within 2 weeks (Chen et al., 2012; Wang et al., 2013b). In addition, animal experiments have shown a positive correlation between the infective viral dose and the average appearance time the tumor, which provided an ideal model for antiviral screening of drugs. In this article, chicken embryo fibroblasts (CEFs) infected with viral stocks of Fu-J (SDAU1005) were used as the target cell to prove that lamivudine could inhibit the replication of ALV-J in cultured cells. We also established an animal infection model using viral stocks of Fu-J (SDAU1005) to confirm that lamivudine could also inhibit the replication of ALV-J and decrease tumor growth *in vivo*. The relationship between the replication-defective virus and its helper virus and tumor processes are also discussed.

## Materials and Methods

### Cells, Virus, and Reagents

The acute transforming Fu-J virus was isolated from an ALV-J associated acute fibrosarcoma of natural cases in crossbreed broilers (Li et al., 2012). The Fu-J (SDAU1005) viral stock used in this study was prepared from cell-free filtrate of acute fibrosarcomas mentioned above, which consisted of both the replication-defective virus Fu-J carrying *v-fps* oncogene and its helper virus, ALV-J strain SDAU1005. The Fu-J (SDAU1005) viral stock could induce fibrosarcomas in chickens rapidly (within 2 weeks) but only gave rise to a mild transformation in cultured CEF (Chen et al., 2012). CEFs were cultured in Dulbecco’s modified Eagle medium (DMEM; GIBCO, Shanghai, China) with 10% FBS at 37°C in 5% CO_2_ atmosphere. Lamivudine was purchased from Glaxo Smithkline (Jiangsu, China) and dissolved in DMEM or deionized water. The ALV-J monoclonal antibody JE9 was kindly gifted from Qin Aijian (Qin et al., 2001). Mouse anti-fps monospecific serum was developed and described previously (Wang et al., 2013b).

### Cell Viability Assay

The CEF viability assay was performed using a Cell Counting Kit-8 (CCK-8; Transgen Biotech, Beijing, China) according to the manufacturer’s instructions. Lamivudine was dissolved and diluted to 0, 1, 2, 3, 4, 6, 8, and 10 μg/ml with PBS buffer. 1.0 × 10^5^ cells suspended in DMEM were seeded in 96-well plates and treated with lamivudine of different concentrations. CCK-8 solution (20 μl) was added to each well to incubate for 5 h, and absorbance at 450 nm was determined. The absorbance at 450 nm of untreated cells was determined to exclude background values, and every detection was repeated three times.

### Virus Infection and Lamivudine Treatment

Chicken embryo fibroblasts were plated in 35-mm dishes preincubated in DMEM with 1, 2, and 4 μg/ml lamivudine. The Fu-J (SDAU1005) viral stock (2.0 MOI of SDAU1005 viruses) was inoculated into cells 12 h later in the presence of lamivudine. After 2-h infection, media with a different concentration of lamivudine were changed and maintained for 6 days. Cell supernatants were collected every day to determine the ALV p27 antigen by ELISA (IDEXX, Beijing, China). Some of the cells were lysed with RIPA buffer (Beyotime, Jiangsu, China) for western blot analysis 6 days later. Meanwhile, RNA was extracted for real-time PCR to quantify the copy numbers of SDAU1005 and Fu-J virus from each group.

### Detection of ALV Reverse Transcriptase Activity

A real-time PCR method modified from the “product-enhanced reverse transcriptase (PERT) method” was used to investigate the inhibitory effect of lamivudine on ALV reverse transcriptase (Pyra et al., 1994; Chang et al., 1997). The commercialized ALV reverse transcriptase used in this study was purchased from Takara (Dalian, China). First, to generate a standardized RNA template, the ALV-J gp85 gene was cloned into pBluescript II-SK(+) vector that carries the T7 promoter sequence to construct the plasmid PSK-gp85. The linear form of PSK-gp85 was digested by *Sac* I restriction enzyme and used to transcribe mRNA using the T7 *in vitro* Transcription Kit (Roche, Switzerland). Next, four groups of reverse transcription reaction were performed by the standardized mRNA template prepared using the commercialized ALV reverse transcriptase (Takara, Dalian, China). For groups 1, 2, and 3, 0.1, 0.2, and 0.4 μl lamivudine water solution (1 mg/ml) was added to each reaction and group 4 served as control, and every reaction was repeated three times. Subsequently, real-time PCR using primers gp85-F and gp85-R listed in **Table 1** was performed to quantify the copy numbers of cDNA (1 μl) from each group. The efficiency of reverse transcription in groups 1, 2, and 3 was analyzed according to CT values of real-time PCR to calculate the relative enzyme activities.

**Table 1:** Primers used in this study.

Primer name	Sequence(5′–3′)	Application gene	Size of fragment
gp85-F gp85-R	5′-AACCAATCATGGACGATGGTA-3′ 5′-TCCAAAGGTAAACCCATATGC-3′	*gp85*	255 bp
fps-F fps-R	5′-GCGAGGGGAACGGACTAATT-3′ 5′-CACGCTGTGACATCCACTTCTT-3′	*v*-*fps*	326 bp
Actin-F Actin-R	5′-GAGAAATTGTGCGTGACATCA-3′ 5′-CCTGAACCTCTCATTGCCA-3′	β-actin	152 bp

### Detection of ALV-p27 Antigen in Intracellular Samples and Cell Supernatants of CEF Infected with Fu-J (SDAU1005) Viral Stock

The ALV-p27 antigen was determined by ELISA according to the manufacturer’s instructions (IDEXX, Beijing, China). Every experiment was repeated three times. For cell supernatant samples, supernatants were collected at the appropriate time and stored at -80°C. For intracellular samples, cells were collected and lysed by rapid freeze-thaw at -80°C and a 37°C water bath.

### Western Blot Analysis to Compare Expression of Fu-J and its Helper Virus

Chicken embryo fibroblasts infected with Fu-J virus were lysed with RIPA buffer, and the protein concentrations were determined using a BCA protein assay kit (Beyotime Biotechnology, Beijing, China). The proteins were denatured by heating and separated on 10% sodium dodecyl sulfate-polyacrylamide gel electrophoresis gels and transferred to a nitrocellulose membrane (Millipore, USA). The membranes were blocked with 5% skimmed milk in PBS containing 0.1% Tween 20 (PBST) for 1 h at room temperature and incubated with mouse anti-fps serum (Wang et al., 2013b) or mAb JE9 antibody (Qin et al., 2001) at 4°C overnight. The expression level of β-actin (Beyotime Biotechnology, Beijing, China) was detected as control. The blots were washed three times with PBST and incubated with the HRP-conjugated secondary antibody (Sigma, USA) for 1 h at room temperature. The blots were washed as above, and positive reactions were detected by enhanced chemiluminescence (ECL) detecting system (Beyotime Biotechnology, Beijing, China).

### Real-time PCR to Compare Expression of Fu-J and its Helper Virus

The expression levels of the SDAU1005 gp85 gene and Fu-J v-*fps* oncogene in CEFs were determined by real-time PCR. Primers used in this study were synthesized by Shanghai Sangon Company (Shanghai, China) and are listed in **Table 1**. Amplification results using primers gp85-F and gp85-R represented the copy number of the replication competent ALV-J SDAU1005, which functions as a helper virus for the Fu-J strain. None of the Fu-J viral genome could be amplified by primers gp85-F and gp85-R because Fu-J virus was defective with the *env* gene deleted. The amplification results using primers fps-F and fps-R represented the copy number of Fu-J. It is notable that the binding sites for the primer fps-F were located in the *gag* region while the binding sites for the primer fps-R were located in the 5′ terminal of *fps* gene, which avoids the non-specific amplification of the cellular *fps* gene. RNA was extracted from infected CEFs using a RNA extraction kit (Omega, USA). Total RNA (1 μg) was reverse transcribed into first-strand cDNA using primeScript RT Master Mix (Takara, Dalian, China) following the manufacturer’s instructions. Diluted cDNA, primers, and SYBR Green Mix (Takara, Dalian, China) were used for the real-time PCR in a final volume of 20 μl. Real-time PCR was performed using the following parameters: 95°C for 30 s, followed by 40 cycles of 95°C for 5 s, and 60°C for 34 s. The expression levels of those genes were normalized with the expression of chicken β-actin mRNA. The analyses of the relative gene expression data were performed by the 2^-ΔΔCT^ method.

To determine the viral loads in chicken plasma, absolute quantitative real-time PCR was established. Two fragments amplified using primers gp85-F/gp85-R and fps-F/fps-R were cloned into PMD-18T vectors to construct the standard plasmids PMD-gp85 and PMD-fps. Those two plasmids were diluted 10^9^ fold to generate the standard curves. Viral RNA was extracted from chicken plasma and reverse transcribed into first-strand cDNA. Real-time PCR was performed using cDNA templates, and viral loads were calculated by the equation generated according to the standard curve.

### Chicken Experiments to Test Antiviral and Tumor Inhibitory Effects of Lamivudine *In vivo*

A total of 160 1-day-old specific pathogen-free leghorn chickens were divided into eight groups and housed in negative pressure-filtered air isolators. The group details are as follows: chickens in groups 1, 2, 3, and 4 and groups 5, 6, 7, and 8 were inoculated with Fu-J virus stock subcutaneously or intraperitoneally, respectively. As described in **Table 2**, chickens in groups 1, 2, and 3 and 5, 6, and 7 were injected intramuscularly with 1, 2, and 4 mg lamivudine dissolved in 400 μl DMEM every day for 7 days. Chickens in groups 4 and 8 were injected with the same volume of DMEM as control. Viral loads of SDAU1005 and Fu-J in plasma of chickens in group 5, 6, 7, and 8 were determined by real-time PCR every week. The occurrence, sizes of tumors, and death rates were observed and recorded every day.

**Table 2:** Groups of the chickens to establish the animal model.

Group	No. chickens	Infection route	Treatment
1	20	Subcutaneous (sc.)	1 mg lamivudine
2	20	Subcutaneous (sc.)	2 mg lamivudine
3	20	Subcutaneous (sc.)	3 mg lamivudine
4	20	Subcutaneous (sc.)	DMEM
5	20	Intraperitoneal (ip.)	1 mg lamivudine
6	20	Intraperitoneal (ip.)	2 mg lamivudine
7	20	Intraperitoneal (ip.)	3 mg lamivudine
8	20	Intraperitoneal (ip.)	DMEM

### Detection of Drug-resistant Mutants in Acute Subcutaneous Sarcomas of Administrated Chickens

The original tumor tissue filtrate was used as a primary viral stock, and isolated viral stock was prepared from subcutaneous tumors collected from chickens infected with primary viral stocks. Both primary viral stock and isolated viral stock were quantified by real-time PCR. Then, CEFs were infected with primary viral stock and isolated viral stock, respectively, with the same dose and cultured in DMEM with 1 μg/ml lamivudine under the same conditions. Simultaneously, infected CEFs cultured in DMEM without lamivudine were used as controls. Cells were maintained for 6 days, and cell supernatants were collected every day to measure the ALV-p27 antigen by ELISA. Viral RNA was extracted on day 7, and both helper virus and defective virus were quantified by real-time RT-PCR. The inhibition ratio of lamivudine was calculated according to viral copy numbers to estimate the inhibitory effect of lamivudine on isolated viral stock. In addition, the sequence coding for reverse transcriptase in the ALV *pol* gene was amplified from both primary viral stock and isolated viral stock to investigate whether mutations of viral drug resistance had occurred during administration.

### Statistical Analysis

The results are presented as the means + the SEMs (*N* = 3). All statistical analyses were performed using SPSS statistical software package for Windows, version 17.0 (SPSS, Inc., Chicago, IL, USA). Statistical comparisons were made using Student’s *t*-test.

### Ethics Statement

The animal experiments were approved by Shandong agricultural university animal care and use committee. The license number was SDAUA-2014-008. Care and maintenance of all chickens were in accordance with the guidelines of the Committee on the Ethics of Animal of Shandong Agricultural University and the biosecurity guidelines. Chickens suffering from tumors grown to a certain degree exceed a diameter of 35 mm or those in bad healthy state due to tumors were sacrificed by well-trained operators to protect animal welfare.

## Results

### Inhibitory Effects of Lamivudine on the Replication of Both ALV-J Associated Acutely Transforming Virus and its Helper Virus *In vitro*

**FIGURE 1:**
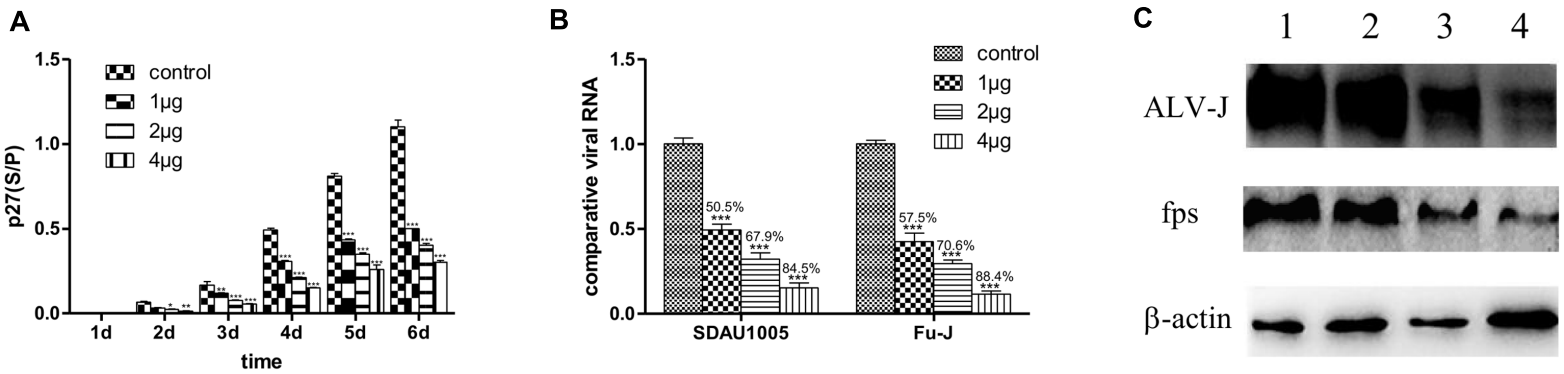
**Inhibitory effects of lamivudine in different concentrations on replication of Fu-J and its helper virus in CEF cells infected with Fu-1 stock. (A)** Comparisons of ALV p27 antigen levels in cell culture supernatants by ELASA. The vertical axis represents the s/p values in ELISA, the cut-off value of positive criteria was 0.2. **(B)** Comparisons of expression level of genomic RNA fragments *gp85* specific to SDAU1005 and *fps* to Fu-J by real-time PCR. The expression levels were normalized with the expression level of chicken β-actin mRNA and performed by using the 2^-ΔΔCT^ method. Differences in the expression level were assessed by Student’s *t*-tests. **(A,B)** Differences were considered significant when *p* < 0.01 (^∗^), highly significant when *p* ≤ 0.01 (^∗∗^) and extremely significant *p* ≤ 0.001 (^∗∗∗^). The error bars represent the SEM. The data are based on the results of three independent experiments. **(C)** Detection of ALV-J gp85 and fps proteins in Fu-J (SDAU1005) viral stock infected CEF cells by western blot analysis. Line 1: control group; lines 2–4: cells treated with 1, 2, 4 μg/mL lamivudine.

**Figure 1** shows that significant inhibitory effects of lamivudine occurred with respect to replication of both Fu-J and its helper virus SDAU1005 in infected CEF cultures. This was based on different criteria in three different assays compared with the control without lamivudine. These criteria and assay included ALV-p27 expression levels in cell culture supernatants by ELSA kit (**Figure 1A**), specific viral genomic RNA fragment levels in cell culture supernatants by real-time RT-PCR (**Figure 1B**), and specific gp85 or fps protein expression levels in cell lysates by western blot analysis (**Figure 1C**). Such inhibitory effects of lamivudine were dose dependent in the concentration range from 1 to 4 μg/ml as indicated in **Figure 1**. To rule out the possibility that the antiviral activity was due to the cytotoxicity of the chemical, a CCK-8 cell viability assay was performed, and this indicated that there was no toxic effect of lamivudine on CEF viability with the concentration used (data was not included), while lamivudine with a concentration exceeding 8 μg/ml resulted in mild damage to CEF.

### Lamivudine Inhibits ALV Reverse Transcriptase Activity

As a nucleotide analog, lamivudine can inhibit HIV reverse transcriptase activity by competition binding with natural substrates for the enzyme. To evaluate the inhibitory effects of lamivudine on ALV reverse transcriptase activity, a real-time PCR method modified from “PERT” was performed. Commercialized ALV reverse transcriptase was employed in this study. Reverse transcriptase reaction systems with different concentrations of lamivudine were performed using 10^5^ copy number of transcribed mRNA. The RT efficiencies of those reactions were analyzed according to the results of real-time PCR CT values. The results illustrated a significant decline of enzymatic activity in the reaction system where lamivudine was present, which indicated a strong inhibitory effect of lamivudine on ALV reverse transcriptase activity (**Table 3**).

**Table 3:** Inhibitory effect of lamivudine on ALV reverse transcriptase activity.

Group	Lamivudine added into RT reaction (μg/mL)	CT value of real-time PCR (mean ±*SD*)^a^	Copy number of RT Products	Relative activity of reverse transcriptase^b^ (%)
1	1	24.72 ± 0.23^B^	2.84 × 10^3^	46
2	2	25.77 ± 0.18^C^	1.21 × 10^3^	24
3	4	27.53 ± 0.19^D^	2.90 × 10^2^	6
4	-	23.97 ± 0.23^A^	5.26 × 10^3^	100

*^a^CT values of real-time PCR followed by different superscript letter was significantly different (*p* < 0.05) based on Duncan’s multiple range test.*

*^b^The relative activity of reverse transcriptase was calculated of copy numbers of RT products in each groups divided by copy number of RT products in group 4.*

### Lamivudine has a Significant Inhibitory Effect on the Growth of Subcutaneous Tumors Induced by Fu-J (SDAU1005) Viral Stock

**FIGURE 2:**
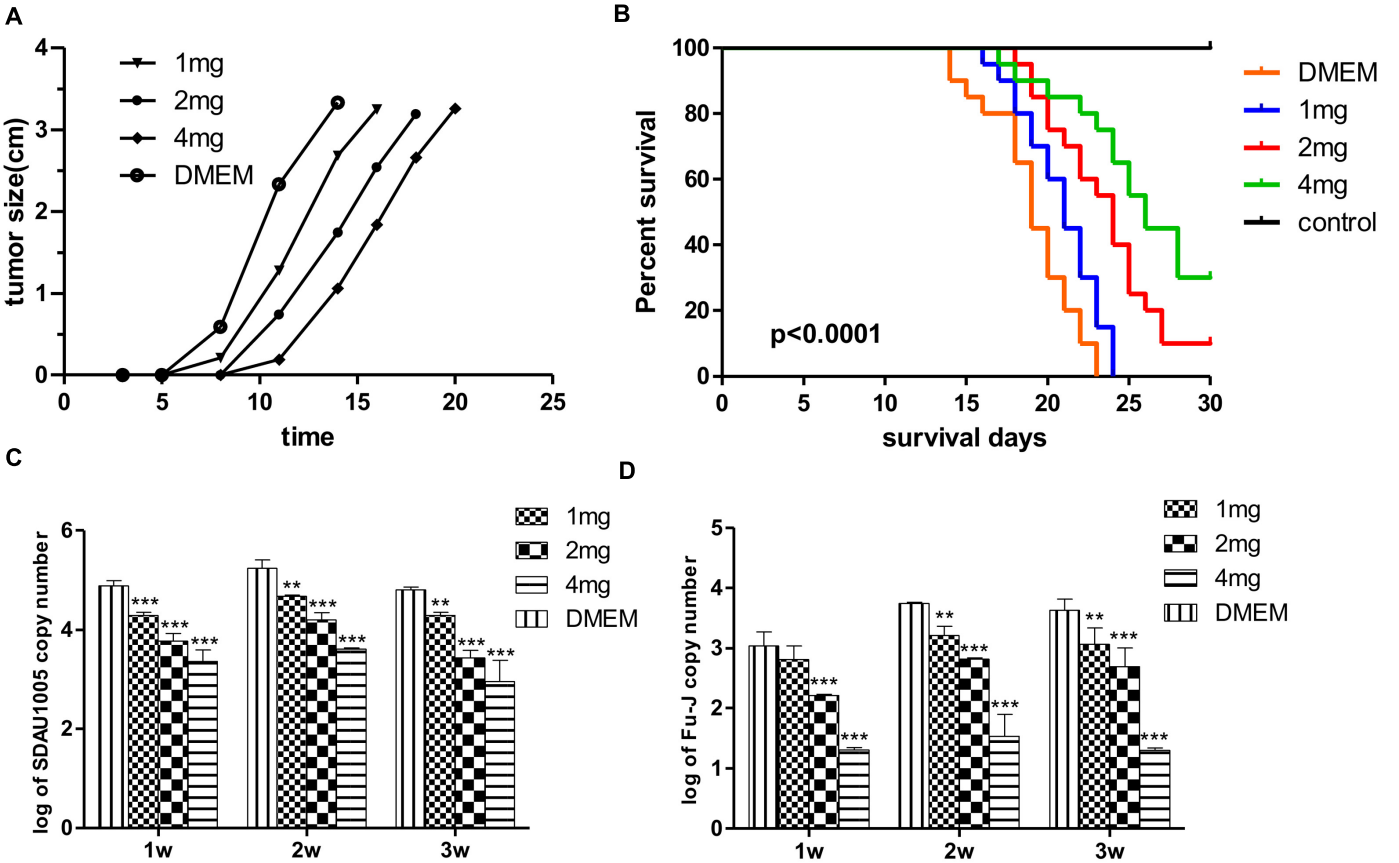
**Effect of lamivudine on Fu-J replication in chickens. (A)** Growth of subcutaneous tumors induced by Fu-J virus in different groups. Administration of lamivudine delayed the tumor occurrence time and slowed down the speed of tumor growth at the early stage. **(B)** Survival plots of chickens infected with Fu-J virus intraperitoneally in different groups. Survival patterns of chickens in different groups showed a significant different from each other. **(C)** SDAU1005 plasma viral loads post-inoculation determined by real-time PCR demonstrated that Lamivudine could inhibit SDAU1005 replication in chickens. **(D)** Fu-J plasma viral loads post-inoculation determined by real-time PCR demonstrated that the replication of Fu-J virus was inhibited due to the reduction of SDAU1005. Differences in the expression level were assessed by Student’s *t*-tests. Differences were considered highly significant when *p* ≤ 0.01 (^∗∗^) and extremely significant *p* ≤ 0.001 (^∗∗∗^). The error bars represent the SEM. The data are representative of the results of three independent experiments.

To determine whether lamivudine had inhibitory effect on the growth of subcutaneous tumors induced by Fu-J, 80 1-day-old chickens were divided into four groups randomly and subcutaneously inoculated with the same dose of Fu-J. Chickens in groups 1, 2, and 3 were injected intramuscularly with 1, 2, and 4 mg lamivudine, respectively, for 7 days, while chickens in group 4 were inoculated with the same amount of PBS and served as a control group. The dynamic growth of subcutaneous tumors induced by Fu-J virus was investigated every day, and chickens were sacrificed when the tumors had grown to a certain extent. The results showed that subcutaneous tumors appeared approximately 5 days after inoculation and measured up to 4 cm in diameter at 15 days with malignant rapid growth (**Figure 2A**). However, subcutaneous tumors occurred later in chickens injected with lamivudine compared with those injected with PBS. Subcutaneous tumors could be detected after 6, 7, and 9 days after inoculation of chickens injected with 1, 2, and 4 mg lamivudine, respectively (**Figure 2A**). Moreover, the tumors grew slowly in chickens without lamivudine injection at an early stage, although tumors grew more rapidly at a later stage compared with those chickens without lamivudine injection. Therefore, it would appear that lamivudine has an inhibitory effect on the early growth of subcutaneous tumors but could not suppress virus replication or tumor growth completely.

### Lamivudine Can Reduce the Mortality of Chickens Inoculated with Fu-J (SDAU1005) Viral Stock Intraperitoneally by Inhibiting Help Virus ALV-J Replication

Another animal model was established for rapid drug screening using the Fu-J virus. Eighty 1-day-old chickens were divided into four groups and inoculated with the same dose of Fu-J virus intraperitoneally. Chickens in groups 5, 6, and 7 were injected intramuscularly with 1, 2, and 4 mg lamivudine every day for 7 days, while chickens in group 4 were injected with the same dose of PBS to serve as a control group. Survival rates were recorded, and viral loads in plasma were determined by real-time PCR every week. The results showed that the median survival for chickens in groups 5, 6, and 7 and the control group was 21, 24, 26, and 19 days, respectively, while the mortality rates were 100, 90, 70, and 100%, respectively (**Figure 2B**). This indicated that lamivudine could prolong the survival time and reduce the mortality rate in chickens infected with Fu-J virus, although the use of drugs could not prevent the growth of abdominal tumors. The real-time PCR results illustrated a significant reduction of SDAU1005 and Fu-J plasma viral loads in chickens injected with lamivudine, and this effect was dose dependent, which could explain the cause of mortality reduction in chickens (**Figures 2C,D**). Furthermore, lamivudine had a more moderate inhibitory effect on Fu-J strain, which carry the v-*fps* oncogene than SDAU1005. This might result from the different inhibitory mechanisms between SDAU1005 and Fu-J. Lamivudine could inhibit replication of SDAU1005 by inhibiting its reverse transcriptase activity, but the inhibition of Fu-J might have resulted from the reduction of SDAU1005, which functions as its helper virus and provides reverse transcriptase and other proteins.

### No Drug-resistant Mutants could be Detected in Acute Subcutaneous Sarcomas of Chickens

**FIGURE 3:**
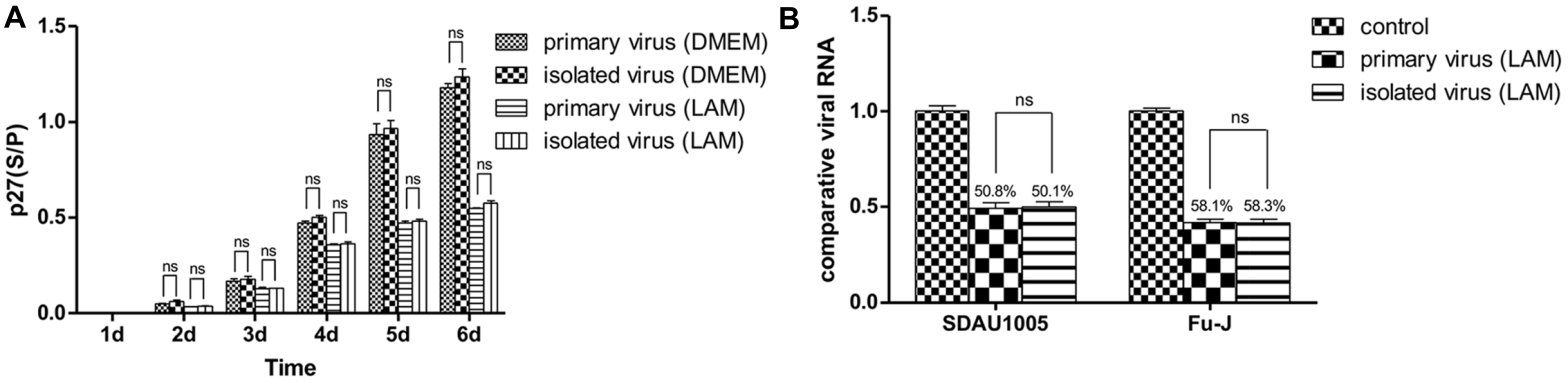
**Chicken embryo fibroblast (CEF) cells were infected with primary viral stock and administrated isolated viral stock respectively and maintained in DMEM with or without 1 μg/mL lamivudine for 6 days, and ALV-p27 antigen was measured from cellular supernatant every day **(A)**.** Viral RNA was extracted on the 7th day and both helper virus and replication-defective virus were quantitated by real-time PCR. Inhibition ratio of lamivudine was calculated to estimate the inhibitory effect of lamivudine on primary viral stock and isolated viral stock **(B)**. Differences in the expression level were assessed by Student’s *t*-tests. The error bars represent the SEM. ns, no significant difference (*p* > 0.05).

Chicken embryo fibroblasts were infected with primary viral stock and isolated viral stock, respectively, and maintained in DMEM with 1 μg/ml lamivudine for 6 days. Helper virus and replication-defective virus were quantified by real-time PCR, and the inhibition ratio of lamivudine was calculated. The ELISA results showed that lamivudine could inhibit the replication of both primary virus and administered isolated virus. However, no significance was observed between primary virus multiplication and administered isolated virus multiplication during cell maintenance (**Figure 3A**). Moreover, the quantitative data also revealed that there was no significance observed between the inhibition ratio of lamivudine on the replication of both helper virus and replication-defective virus (**Figure 3B**). In addition, the viral sequence coding for reverse transcriptase in the ALV *pol* gene was amplified from primary virus and isolated virus, and sequences were compared. However, no regular mutations related to drug resistance were observed. Above all, there was no detectable drug resistance mutations that had occurred in the viral stock prepared from subcutaneous tumors collected from chickens infected with primary viral stocks.

## Discussion

Throughout history, ALV, especially ALV-J, has caused great economic losses for the poultry husbandry in China (Gao et al., 2010). Much research has been conducted to discover or design agents for antiviral therapy (Qian et al., 2014; Wei et al., 2015). However, no effective vaccine or drug agents are available at present due to the high mutation rates of ALV-J. It appears that surveillance and elimination of infected chickens are the most fundamental and effective way to control ALV-J since it is mainly transmitted vertically from generation to generation (Weiss and Vogt, 2011). Drug administration could also inhibit virus replication and transmission to virus-free chickens, which could accelerate virus control objectively.

Lamivudine is a typical antiviral agent, a pyrimidine nucleoside, which can be phosphorylated in cells and inserted into viral DNA in cyclic adenosine phosphate forms, leading to the termination of DNA extension (Balzarini, 1994). In addition, lamivudine has a strong inhibitory effect on HIV by directly inhibiting HIV reverse transcriptase activity (Tisdale et al., 1993; Gray et al., 1995; Anderson, 2002). Currently, lamivudine has been widely used for the treatment of hepatitis B and HIV with a mature synthetic technique (Bernasconi and Battegay, 1998; Anderson, 2002; Fung et al., 2011). Phylogenetic comparison of HIV and ALV reverse transcriptases showed a 39.6% identify. Given that reverse transcriptase played important roles in ALV replication and infection, we considered lamivudine may also have an inhibitory effect on ALV; however, to the best of our knowledge, no research had been conducted in this area till date.

In this study, a series of experiments were conducted in CEF cultures and 1-day-old chickens inoculated with an acute transforming viral stock Fu-J (SDAU1005). In fact, this viral stock consisted of two types of viruses: the replication-defective virus Fu-J carrying the *v-fps* oncogene and its helper virus ALV-J SDAU1005 (Chen et al., 2012; Wang et al., 2013b). Fu-J (SDAU1005) viral stock was employed in this study because it can rapidly induce fibrosarcomas in chickens within 2 weeks. In addition, there was a positive correlation between occurrence time and growth speed of tumors and the viral infection dose. This makes it more convenient for us to evaluate the inhibitory effect of lamivudine on ALV-J in chickens by recording and analyzing several parameters within a relatively short period. The results in cell cultures demonstrated that lamivudine could inhibit the replication of both SDAU1005 and Fu-J viruses and was dose dependent in the concentration range of 1–4 μg/ml. Meanwhile, the animal experiment data showed that lamivudine could slow down the growth and decrease the sizes of the tumor, while prolonging the survival time and delaying the mortality of infected chickens at an early stage by decreasing the viral loads of both SDAU1005 and Fu-J virus carrying the v-*fps* oncogene in chicken plasma. Furthermore, the mechanism of the inhibitory effect was elucidated. We showed that lamivudine had an inhibitory effect on ALV reverse transcriptase reverse activity. Therefore, it seems that lamivudine could inhibit ALV-J replication by competing with normal nucleotides for reverse transcriptase binding and inhibited cDNA transcription and extension, with a mechanism similar to that of HIV inhibition. Given that the sequence of the *pol* gene, which encoded reverse transcriptase, was conserved in different subgroups of ALV, we consider that lamivudine could inhibit all subgroups of ALV strains other than ALV-J. Those results suggested lamivudine might serve as a potential antisubgroup J ALV therapy.

It is notable that the replication of helper virus ALV-J SDAU1005 strain was blocked by lamivudine through inhibition of the reverse transcriptase enzyme activity. However, the replication-defective virus Fu-J could not encode reverse transcriptase due to a deletion mutation of the *pol* gene with the viral genome structure as follows: 5′ LTR-Δ*gag*-*fps*-Δ*pol*-Δ*env*-3′ LTR. Thus, it would be expected to rely entirely on the helper virus for *gag-, pol-*, and *env-*encoded protein functions to replicate and infect other host cells. We speculate that the inhibitory capability of Fu-J virus in CEF cultured cells and in chickens inoculated with Fu-J (SDAU1005) was due to the inhibitory effect of lamivudine on SDAU1005, which functions as a helper virus of Fu-J. This study demonstrates that the level of Fu-J virus replication correlated with that of SDAU1005, which provided an insight into the relationships between the replication-defective virus and its helper virus, as well as with tumor processes.

Finally, it is important to highlight that lamivudine treatment cannot kill the virus completely. In fact, when used alone for HIV therapy, drug-resistant mutants can develop quickly due to the high mutation rate of HIV during virus replication (Boucher et al., 1993; Tisdale et al., 1993; Gu et al., 1994; Smith et al., 1994). Therefore, highly active antiretroviral therapy (HAART) using several combinations of drugs at the same time is usually carried out for HIV treatment. In this study, the growth rate of acute sarcoma was obviously increased at the late stage, highlighting suspicion of whether the virus had mutated to resist drug therapies. However, our experimental data showed that even lamivudine at low concentration could still inhibit the replication of the virus from administered chickens, and the sequence of the *pol* gene coding for reverse transcriptase did not illustrate any regular mutations, which contributed to drug resistance. We conclude that no detectable drug-resistant mutants developed in infected chickens treated with lamivudine, during our study period. The increase in tumor growth might result from unregulated tumor cell proliferation in chickens. The same method was also performed to investigate whether drug-resistant mutants had developed in the cell supernatant of cultured CEFs maintained in DMEM with added lamivudine. The results demonstrated that no drug-resistant mutants were detected in cell supernatant. However, our latest data showed that when the viral stock was passaged to the 38th generation in cultured CEFs maintained in DMEM with lamivudine added, the replication of virus could not be inhibited by lamivudine, which implied that drug-resistant mutants could develop *in vivo*, and we intend to publish related data at a later date. The possibility of drug-resistant mutants should be considered during the treatment of ALV using lamivudine. Therefore, it is necessary to use other drugs in a similar way to that conducted in “HAART,” to avoid the risk of virus mutation and obtain a better therapeutic effect. It is important to emphasize that eradication of ALV in infected chickens is one of the most fundamental measures for ALV prevention and control. Drug administration is just a supplementary means of controlling virus transmission and accelerates the purification process.

## Conflict of Interest Statement

The authors declare that the research was conducted in the absence of any commercial or financial relationships that could be construed as a potential conflict of interest.
